# Research Progress of Population Pharmacokinetic of Metformin

**DOI:** 10.1155/2022/4071111

**Published:** 2022-12-19

**Authors:** Xiaohu Wang, Jin Tang, Chaozhuang Shen, Xingwen Wang, Hua Hu, Haitang Xie

**Affiliations:** ^1^Anhui Provincial Center for Drug Clinical Evaluation, Yijishan Hospital of Wannan Medical College, No. 2, Zheshan West Road, Jinghu District, Wuhu 241000, China; ^2^Wannan Medical College, No. 22, Wenchang West Road, Yijiang District, Wuhu 241000, China

## Abstract

Metformin is commonly used as first-line treatment for T2DM (type2 diabetes mellitus). Owing to the high pharmacokinetic (PK) variability, several population pharmacokinetic (PPK) models have been developed for metformin to explore potential covariates that affect its pharmacokinetic variation. This comprehensive review summarized the published PPK studies of metformin, aimed to summarize PPK models of metformin. Most studies described metformin pharmacokinetics as a 2-compartment (2-CMT) model with 4 study describing its pharmacokinetics as 1-compartment (1-CMT). Studies on metformin PPK have shown that obesity, creatinine clearance (CL_Cr_), gene polymorphism, degree of renal function damage, and pathological conditions all have a certain impact on the PK parameters of metformin. It is particularly important to formulate individualized dosing regimens. For future PPK studies of metformin, we believe that more attention should be paid to special populations.

## 1. Introduction

Metformin is a full-course drug for the treatment of type 2 diabetes mellitus (T2DM), and its main pharmacological effect is to lower blood sugar by reducing hepatic glucose output and improving peripheral insulin resistance [1, 2]. Metformin is recommended in the guidelines for the diagnosis and treatment of diabetes formulated by many countries and international organizations as the first-line drug for controlling hyperglycemia in T2DM patients and the basic drug in drug combination [3].

Metformin is mainly absorbed and distributed in intestinal epithelial cells and hepatocytes, and is transported by organic cation transporters 1(OCT1), organic cation transporters 3 (OCT3), and novel organic cation transporters 1 (OCTN1) [4, 5]. After absorption, it is transported by MATE (mammal multidrug and toxin extrusion protein), and finally discharged by kidney and urine in prototype [6].

Studies have shown that the plasma concentrations of metformin vary greatly among humans, and about 1/3 of patients cannot achieve satisfactory hypoglycemic effects [7, 8]. Gene polymorphism, renal function, obesity, and other factors may be important factors for individuals' response to metformin [9, 10]. Christensen et al. [11]. showed that the plasma steady-state trough concentration of subjects taking the same dose of metformin was 54~4133 ng/ml, suggesting that there may be large individual differences in the pharmacokinetic behavior of the drug, and the dosage of some patients needs to be adjusted to achieve satisfactory therapeutic effects.

Population pharmacokinetic combines classical pharmacokinetic principles with population statistical models, and is a research method to study the causes and correlations of drug concentration differences between individuals. Currently, it is used in a variety of drugs, including anesthetic drugs [12], anti-infective drugs [13, 14], and antituberculosis drugs [15] and other drug regimen formulations, and the expected effects are obtained.

The population pharmacokinetics study of metformin can explore the population characteristics of metformin and its related influencing factors, and by simulating the drug exposure under different dosing schemes, make the clinical dosing scheme reasonable and effective, improve the curative effect and reduce the occurrence of adverse reactions. Thus, this review aims to compile all published PPK models of metformin, focusing on PK parameters and the influence of covariates to optimize treatment in order to provide reference for clinical rational use of metformin and its population pharmacokinetics.

## 2. Methods

### 2.1. Literature Search Strategy

The EMBASE and PubMed databases were searched (up to June 2022) using the following terms: “metformin” AND (“population pharmacokinetics model” OR “pharmacometrics” OR “pharmacokinetic model” OR “nonlinear mixed effect model” OR “NONMEM” OR “model”). The reference lists from the relevant studies were analyzed for additional literature.

### 2.2. Inclusion Criteria and Exclusion Criteria

Studies were included in this systematic review if they met the following criteria:

(1) Human studies; main indication was T2DM; both patients and healthy subjects; (2) metformin as study drug; providing PPK analyses; (3) employing a nonlinear mixed effect modeling approach. (4) Not written in English; reviews conducted in vitro and animal studies were excluded.

### 2.3. Data Extraction

The following information were extracted from each included study:

1. The study characteristics. e.g. authors, year, study size, type of study (Prospective/Retrospective), number of participants, and dosage regimens
2. The characteristics of the target population, e.g. patients or healthy subjects, male/female, race, collected samples, age, and weight range; and
3. The information on PPK analyses, e.g. structural and statistical models, data analysis software, covariates, parameter estimates, sampling schedule (sparse sampling/intensive sampling), estimation method (First-order conditional estimation with interaction, FOCE-I; First-order conditional estimation, FOCE), interindividual variability (IIV), residual variability (RV), and model evaluation approaches

## 3. Results

### 3.1. Study Identification

**Figure 1:**
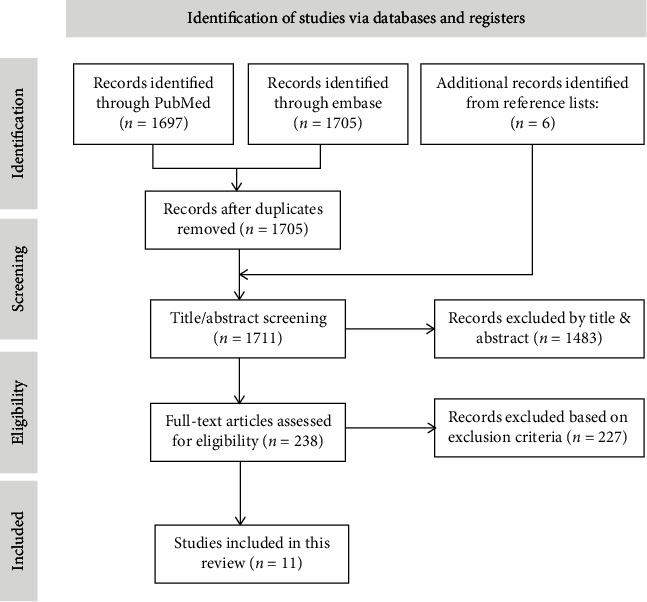
The selection process of the studies included in the systematic review.

A total of 1721 articles were identified from PubMed and EMBASE of relevant studies. After preliminary reading and repeated review, 1483 articles were not deemed to be in accordance with the inclusion criteria. In the remaining 238 articles, 4 record was a review article, and 223 articles did not provide PPK analyses or did not use NONMEM [16, 17]. Finally, only 11 studies were included in this systematic review. The specific process is shown in Figure 1.

### 3.2. Study Characteristics

All included studies were published between 2006 and 2020. The characteristics of each study are summarised in Table 1. Among the 11 studies, 4 were retrospective studies and 7 were prospective studies. Almost all of these studies were conducted in adults, with only one in adolescents. Of these, 4 were in patients with type 2 diabetes (2 special population studies: pregnant women and patients receiving haemodialysis), 4 are in healthy volunteers, and the remaining 2 include T2DM patients and healthy volunteers; the overall aim of most population pharmacokinetic studies of metformin has been to identify factors influencing metformin pharmacokinetics and to provide population estimates of the PK parameters. The number of participants in each study ranged from 12 to 336.

### 3.3. Population Pharmacokinetic Models, Pharmacokinetic Parameters, and Covariates

**Figure 2:**
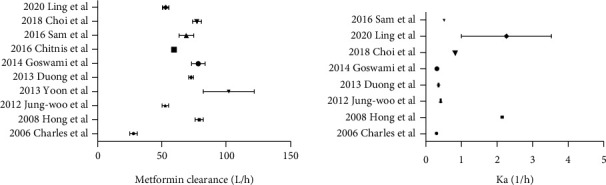
Metformin clearance and Ka included in the study.

The reported sampling schedule, model structure, PK parameters, Estimation method, covariates, and conclusion are summarized in Table 2. All metformin PPK studies included in this review were conducted with NONMEM® software. In terms of disposition, 7 studies described metformin pharmacokinetics using a 2-CMT models; FOCE was the method most frequently employed, while some studies used the FOCE-I method. Many factors were investigated in the process of modeling, such as age, gender, genetic variants in transcription factors, liver function (ALT), creatinine clearance (CL_Cr_), and weight. Three studies used CL_Cr_ as a covariate, and CL_Cr_ was positively correlated with CL/F. One study included total body weight (TBW) as a covariate, and TBW was positively correlated with CL/F too. One study identified the strongest association of SP1 variants with metformin, PK, and PD. In addition, a study showed that the OCTN1-917C > T gene variant and the OCT2-808 G > T gene polymorphism can be used to determine the optimal dose of metformin. All the models were internally validated by a visual predictive check (VPC) [18–23], normalized prediction distribution error (NPDE) [19, 24], boot-strap analysis [18–21, 23–25], diagnostic plots [18–21, 23–27], and the prediction error test [23, 28]. In terms of pharmacokinetic parameters, except for studies in special populations, CL/F is basically between 50-80 l/h (Figure 2). Ka generally ranged from 0.4 to 0.6, and the Ka in the study of Li et al. [24, 25] differed from the rest, possibly due to the small number of subjects included and the number of blood samples collected. However, in patients with late pregnancy, Ka was slightly lower and may have to significant anatomical, physiological, and biochemical differences resulting in maternal pharmacokinetic changes. For the random effect model, IIV was modeled using exponential error model in most studies (Table 3). As for RV, three studies used combined additive and proportional error model. The rest were modeled using additive (*n* = 4), exponential (*n* = 1), and proportional (*n* = 2) error model. The fixed effect parameters of CL/F and V/F are summarized in Table 4.

## 4. Discussion

**Figure 3:**
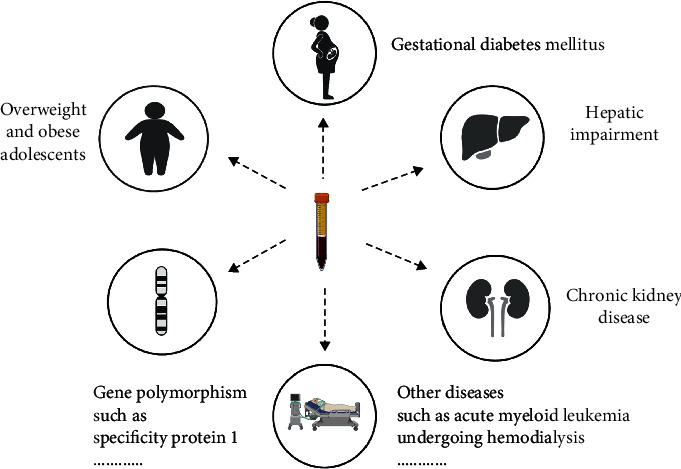
Special populations in which metformin therapeutic drug monitoring (T2DM) may be warranted. Created with http://BioRender.com.

Although metformin has many years of clinical application experience, there are significant individual differences in the efficacy and adverse reactions of metformin. Metformin not only has appetite loss, nausea, diarrhea, and gastrointestinal reactions, but also has serious adverse reactions such as lactic acidemia and ketosis. It is an effective measure to reduce the adverse reactions and improve the curative effect by reducing the dosage. Many studies have put forward effective opinions that controlling the blood concentration of metformin below 5 mg/L can reduce the risk of lactic acid poisoning. Diabetic patients are often accompanied by a variety of complications and require a combination of drugs to achieve satisfactory therapeutic effects. Previous studies have shown that drug transporter gene polymorphisms and the interaction of drugs of different properties may change the PK parameters of metformin, thereby affecting its clinical efficacy, and even causing serious complications or adverse reactions. Dosing regimens can be optimized through population pharmacokinetic studies that provide quantitative evidence of efficacy and safety. Currently, PPK models for metformin have covered a wide range of disease patient populations, with most studies incorporating Bayesian feedback and some using Monte Carlo simulations for optimization of metformin dosing regimens. In the 11 PPK studies collected in this study, the main PK parameters were CL and Vd. Of these, CLcr was the main influence on clearance, while the main influence on Vd was body mass (Table 4). In addition, the study of metformin PPK model should consider the special population as the object such as in patients with hepatic impairment, chronic kidney disease (stage 2, 3(a), 3(b), and 4) [29–32], acute myeloid leukemia, pregnant [33–35], elderly [36], overweight and obese adolescents [37], and so on [38–40] (Figure 3). For example, OCT1 is a major determinant of metformin uptake by hepatocytes, and genetic polymorphisms in OCT1 are associated with variability in metformin PK; promoter variants of the transporter proteins MATE1 and MATE2K, which determine metformin excretion into the urine, are also associated with metformin disposition and response, and understanding variability in metformin response and disposition is important for the rational use of metformin. Stocker et al. [41] studied the effect of novel promoter variants in the gene encoding the MATE transporter on the pharmacokinetic and pharmacodynamic parameters of metformin in healthy volunteers and showed that diabetic patients carrying the MATE1 rs2252281 (T > C) mutation gene had better efficacy on metformin than wild type. Song et al. [42] showed that metformin renal tubular excretion was mainly affected by the OCT2 (586C > T, 602C > T, and 808G > T) mutation, and plasma concentrations of metformin were higher in mutant subjects. In pregnant women, the increased clearance of metformin during pregnancy is due to enhanced renal clearance. From a pharmacokinetic perspective, this may require a metformin dose increase of ≥20% to maintain the given therapeutic effect [33], which can be simulated by population pharmacokinetic methods of dosing. Of concern is that lactate clearance is significantly limited in patients with severely impaired liver function, and some studies suggest that metformin should be avoided in patients with serum transaminases above 3 times the upper limit of normal or with severe hepatic insufficiency. Studies on different populations suggest that the PK parameters of metformin vary among populations, and although some studies have given specific recommendations for dosing regimens, considering the small sample size in the study populations, future PPK studies of metformin should pay more attention to special populations, and more and more extensive studies with large samples are needed to validate them in order to make specific recommendations on the use of metformin in special populations. The dosing of metformin in special populations should be recommended. Besides, previously published models, as well as future models, should be evaluated externally for a more accurate description of models' performance. More importantly, the clinical background needs to be fully considered when including covariates. Although we managed to cover a series of important articles on the popPK analysis of metformin, certain limitations still exist. On the one hand, the pharmacokinetic and pharmacodynamic aspects of metformin have been well studied in terms of genetic polymorphisms and ethnic differences, while population pharmacokinetics have been rarely addressed. For example, there are also studies showing that metformin monotherapy is more effective in Hispanic and non-Hispanic whites compared to non-Hispanic blacks [43]. Mean CL/F and Q/F estimates were significantly higher in African Americans compared to European Americans and Asian Americans. A 26% increase in dose should be considered for African Americans to achieve similar metformin exposure as European Americans [20]. On the other hand, there are aspects of interest regarding drug-drug interactions between metformin and other drugs, as well as new potential in therapeutic treatments beyond glycemic control, especially in the prevention of cancer and treatment of fertility problems in polycystic ovary syndrome, which are not discussed in this review. Apart from that, there were also a small number of population pharmacokinetics of metformin that did not use NONMEM and were not included for correlation analysis. Finally, the review could provide information on the utilized model structure, population pharmacokinetic parameters, influential covariates, as well as the degree of pharmacokinetic variability. We hope to provide some reference for future population pharmacokinetic studies of metformin.

## 5. Conclusion

This review summarizes information on PPK of metformin. Pharmacokinetic parameters of metformin are affected by many factors, including respects of transporter gene polymorphism, kidney function, body weight, and physiological function. However, there are few studies on metformin in populations with special metabolic profiles, such as obese adolescents, patients with gestational diabetes mellitus, liver insufficiency, and it is of great clinical value to study the population pharmacokinetics of metformin in special populations. In conclusion, novel or potential covariates represent an important direction for further research; metformin PPK model in special population patients is still lacking and is recommended.

## Figures and Tables

**Table 1:** Demographics of the published population pharmacokinetic studies.

Study	Site	Male/female	Age (year)	Weight (kg)	Dose regimen (mg/day)	Assay	Subject characteristics	Type of study
2006 Charles [28]	Australia	0/27	32 (26–43)	88 (50–150)	2000 (1000–3000) mg	HPLC	T2DM with late pregnancy	Prospective
2008 Hong [25]	America	3/9	56 (45-63)	89(70-127)	500 mg,5d, bid; 850,5d, bid	LC/MS/MS	T2DM	Prospective
2012 Jung-woo [18]	Korea	42/0	26(21-31)	69(61-78)	Single oral 500 mg	LC-MS/MS	Healthy	Prospective
2013 Yoon [22]	South Korea	96/0	22.41(19-31)	67.74(53.1-95.6)	Single oral 500 mg	HPLC-UV	Healthy	Retrospective
2013 Duong [19]	Australia	NA	A: 65 (39–86) B: 27 (19–40) C: 23 (18–47)	A: 86 (53–165) B: 70 (53–103) C: 58 (41–88)	A: 1,500 (250–3,000) B: 1,000 (500–2,000) C: 500 (500–1,000)	HPLC-UV	Healthy; T2DM A: T2DM, CKD B: Healthy C: Healthy	Retrospective
2014 Goswami [20]	America	NA	46 (18-90)	86.4 (34-212)	Healthy:1850/850 mg T2DM: NA	NA	Healthy:T2DM 102 : 133	Prospective
2016 Chitnis [26]	America	159/177	25.0 (18–50)	64.7 (45.0–111.1)	NA	LC-MS	Healthy	Retrospective
2016 Sam [23]	America	NA	11.2 (7.7-13.5)	77 (50.5-118)	1000 mg, bid	UPLC	Severely obese children with insulin resistance.	Prospective
2018 Choi [21]	South Korea	36/0	23.9(20.0-42.0)	176(169.1-183.5)	NA	LC/MS/MS	Healthy	Prospective
2020 Sinnappah [27]	Australia	3/2	70(61-82)	86(78-102)	500 mg, three times a week	UPLC-MS/MS	T2DM patients undergoing hemodialysis	Prospective
2020 Ling [24]	China	85/40	56 (27–83)	75 (51–113)	2000 (1000–2000) mg	HPLC-UV	T2DM	Prospective

NA: Represents the missing value, which is not mentioned in the article; T2DM: Type2 diabetes mellitus; HPLC: High-performance liquid chromatography; CKD: Chronic kidney disease.

**Table 2:** A summary of published population pharmacokinetic studies of metformin.

Study	Sampling schedule	Structure model	Estimation method	Subject/samples	Pharmacokinetic parameters	Covariates/conclusion
2006 Charles [28]	Sparse sampling	2-CMT	FOCE	27/NA	CL = 28.0 L/h/70 kg; V2 = 190 L/70 kg; Ka = 0.3/h	No dosage adjustment is warranted.
2008 Hong [25]	Intensive sampling	1-CMT	FOCE	12/180	CL = 79.0 L/h; CL/F = 79.0 · (CLCR/80)^0.822^; V = 648 L (13.8%); Ka = 2.15/h	CL_CR_ significantly influenced metformin CL/F
2012 Jung-woo [18]	Intensive sampling	1-CMT	FOCE	42/504	CL = 52.6 L/h (4.18%); CL/F = 52.6 (CR_CL_/106.5)^0.782^ Vd = 113 L(56.6%) Ka = 0.41/h	CL_CR_ significantly influenced metformin
2013 Yoon [22]	Intensive sampling	1-CMT	NA	96/NA	CL/F = 102 ± 34.5 L/h; Vd = 447 ± 214 L	OCT2-808G > T; OCTN1-917C > T
2013 Duong [19]	NA	2-CMT	FOCE-I	A:120/NA B:16/NA C:169/NA	CL = 72.7; Vc = 149 L; Ka = 0.35/h	CL_CR_; TBW The concentrations of metformin do not exceed 5 mg/l
2014 Goswami [20]	Sparse sampling	2-CMT	FOCE-I	235/2383	CL/F = 78.4 L/h; Vc/F = 76.8 L; Vp/F = 413 L; Ka = 0.31/h	Five variants in specificity protein 1 (SP1)
2016 Chitnis [26]	Intensive sampling	2-CMT	NA	336/NA	CL/F = 59.5 L/h; V2 = 211 L; Ka = 0.636/h	Not significant
2016 Sam [23]	Intensive sampling	1-CMT	FOCE	28/336	CL/F = 68.1 L/h; Vd = 28.8 L K_a2_ = 0.324/h	Body weight; SLC22A1
2018 Choi [21]	Intensive sampling	2-CMT	FOCE-I	36/NA	CL/F = 76.7 L/h; Vc/F = 180 L; Vp/F = 109 L; Ka = 0.83/h	Not significant
2020 Sinnappah [27]	Sparse sampling	2-CMT	FOCE	5/184	CLNR/F = 0.49 L/h; Ka = 0.51/h; V2 = 17.5 L; V3 = 48.6	The concentrations of metformin do not exceed 5 mg/l
2020 Ling [24]	Sparse sampling	2-CMT	FOCE-I	125	CL/F = 53.0 L/h; Ka = 1.47/h	Kidney function; eGFR

1-CMT, one-compartment model; 2-CMT, two-compartment model; NA, Represents the missing value, which is not mentioned in the article; FOCE, First-order conditional estimation; FOCE-I, First order conditional estimation with inter and intrasubject variability interaction; CL_CR_, Creatinine clearance; V2, Maternal volume of distribution; CL, total body clearance; Vd, Apparent volume of distribution; Vc/F, Central volume of distribution; Vp/F, Peripheral volume of distribution; Ka, Absorption rat; K_a2_, absorption rate constant at the second absorption site.

**Table 3:** Details of the covariate building process.

Study	Tested covariate	Covariate selection method	Statistical level for selecting significant covariate (*p* value)	IIV		RV	
				Type	Value	Type	Value
2006 Charles [28]	Age, weight	(1) Forward stepwise inclusion (2) Backward elimination	(1) *p* <0.01	CL/F: Logarithmic	17.1%	Additive	SD =0.32 mg/L
2008 Hong [25]	Age, weight, CL_CR_	Univariate analysis	*p* <0.01	Exponential	23.0%	Additive	209 ng/mL
2012 Jung-woo [18]	Gender, age, height, weight, CLCR, total bilirubin, haemoglobin	Forward stepwise inclusion	*p* <0.01	Additive	27.9%	Additive	23.1 ng/mL
2013 Yoon [22]	Weight, age, BSA, ALT, AST, ALP CL_CR_	(1) Forward stepwise inclusion (2) Backward elimination	(1) *p* <0.05 (2) *p* <0.01	Exponential	*ω* ^2^ = 0.08	Proportional	*σ* ^2^ = 0.0291
2013 Duong [19]	TBW, CL_CR_, LBW,57 single-nucleotide polymorphisms (SNPs) of metformin transporters (OCT1, OCT2, OCT3, MATE1 and PMAT)	(1) Forward stepwise inclusion (2) Backward elimination	(1) *p* <0.05 (2) *p* <0.01	Exponential	34.1%	Combined	NA
2014 Goswami [20]	SNPs	Stepwise covariate analysis	*p* <0.01	NA	50%	Combined	NA
2016 Chitnis [26]	Race, metformin country source Body weight, sex, and age	(1) Forward selection using likelihood ratio test (2) Backward elimination	(1) *α* =0.05 (2) *α* =0.01	Combined	22.5%	Additive proportional	*σ* = 0.0359 *σ* = 3.59
2016 Sam [23]	Age, body weight, height, body mass index, sex, race, ethnicity, genotype group and so on	(1) Univariate analysis (2) Stepwise backward deletion	(2) *p* <0.001	Exponential	*ω* ^2^ = 0.0811	Additive	SD = 1.14 mg/L
2018 Choi [21]	Age, weight, Height, and creatinine clearance	(1) Forward selected using likelihood-ratio tests (2) Backward elimination	*p* <0.05	Combined	19.6%	Proportional	*σ* = 0.254
2020 Ling [24]	Weight, BMI, age, eGFR and the genotype of the transporters	(1) Forward stepwise inclusion (2) Backward elimination	(1) *p* <0.05 (2) *p* <0.01	Exponential	16.25%	Exponential	34.64%

Body surface area (BSA), creatinine clearance (CL_CR_), liver function [alanine aminotransferase (ALT), aspartate aminotransferase (AST), and alkaline phosphatase (ALP)]; Total body weight (TBW), lean body weight (LBW), SNPs: Single-nucleotide polymorphisms (SNPs).

**Table 4:** Comparison of CL/ F and V/F in the PPK study of metformin.

Study	CL/F	Vd/F (Vc/F)
2008 Hong [25]	CL/F = 79.0(CL_CR_/80)^0.822^	NA
2013 Yoon [22]	CL/F = 136(1 − 0.248 × *θ*_OCT2_)(1 − 0.234 × *θ*_OCTN1_)	V/*F*_*TV*_ = 112(1 + 0.0183 × *θ*_BW_)
2012 Jung-woo [18]	CL/F = 52.6(CL_CR_/106.5)^0.782^	NA
2013 Duong [19]	CL/F = (*θ*_CL_ × (CL_CR_/6)) × e^PPVCL^	V1/*F* = (*θ*_V1_ × (TBW/70)) × e^PPVV1^
2014 Goswami [20]	CL/F = 78.5(1 + *θ*_CR_CL__(CL_CR_ − 112))(1 + *θ*_Ethnicity,CL_)(1 + *θ*_rs784888_(SP1 − 0))	V_C_/F = 76.8(1 + *θ*_rs555754_)(OCT3_rs555754_ − 0)(1 + *θ*_WT_(WT − 75))(1 + *θ*_rs316019_)
2020 Sinnappah [27]	$\text{CL}/\text{F}=\left(\theta_{\text{CLR}/\text{F}}\left(\frac{\text{CLcr}/\left(\text{L}/\text{h}\right)}{6\left(\text{L}/\text{h}\right)}\right)\right)+\left(\theta_{\text{CLNR}/\text{F}}\times \theta_{\text{F}\_\text{CLNR}\_\text{ID}1}\right)$	NA

## Data Availability

The data that support the findings of this study are available on request from the corresponding author.
